# Interleukin-1 in cerebrospinal fluid for evaluating the neurological outcome in traumatic brain injury

**DOI:** 10.1042/BSR20181966

**Published:** 2019-04-12

**Authors:** Yingming Yue, Chongzhi Shang, Huajiang Dong, Kun Meng

**Affiliations:** 1 Department of Infectious Disease, Beijing Tiantan Hospital of Capital Medical University, Beijing 100070, China; 2 Department of Neurosurgery, Neurological Hospital, Affiliated Hospital of Logistics University of Chinese People’s Armed Police Forces, Tianjin 300161, China; 3 Logistics University of Chinese People’s Armed Police Forces, Tianjin 300192, China

**Keywords:** cerebrospinal fluid, differential expressed proteins, interleukin-1, traumatic brain injury

## Abstract

**Objective** Severe traumatic brain injury (TBI) is associated with unfavorable outcomes secondary to injury from activation of the inflammatory cascade, the release of excitotoxic neurotransmitters, and changes in the reactivity of cerebral vessels, causing ischemia. Inflammation induced by TBI is complex, individual-specific, and associated with morbidity and mortality. The aim of the present study was to discover the differentially expressed cerebrospinal fluid (CSF) proteins and identify which can improve the clinical outcomes in TBI patients.

**Methods** In the present study, we reported 145 patients with TBI and found the change in patients’ leukocytes in serum and interleukin-1 (IL-1) in CSF, which strongly correlated with the neurological outcome. In terms of results of leukocytes in blood and IL-1 in CSF, we retained the patient’s CSF specimens and conducted a proteomic analysis.

**Results** A total of 119 differentially expressed proteins were detected between samples of TBI and the normal, which were commonly expressed in all samples, indicating the differentially expressed proteins. When the patients’ Glasgow outcome score (GOS) improved, IL-1 was down-regulated, and when the patients’ GCS score deteriorated, IL-1 was up-regulated accompanied with the progression in TBI.

**Conclusion** The differentially expressed proteins in CSF may be the novel therapeutic targets for TBI treatment. The leukocytes in blood samples and the IL-1 in CSF may be two important indicators for predicting the prognosis of TBI patients.

## Introduction

### Traumatic brain injury

Traumatic brain injury (TBI) is a leading cause of morbidity and mortality in both the civilian and military settings [1,2]. Like many other forms of injury, TBI is associated with an acute inflammatory response that drives, and in turn is likely driven by further damage dysfunction [3–6]. The complexity of inflammation is daunting and to date there have been no effective therapies that modulate inflammation in TBI [1,7]. A previous study reported that an estimated 1.7 million people stain a TBI annually and TBI is a contributing factor to one-third (30.5%) of all injury-related deaths in the United States [8]. Despite research leading to innovative treatments and standardized care, TBI-related morbidity remains a major cause of disability in the United States with an estimated 5.3 million people living with long-term cognitive and psychological impairments each year [2]. Although improvements in post-TBI mortality have been seen in recent years, morbidity following a severe TBI remains extremely high [2,8].

Aseptic inflammatory response plays an important role in the progress of patients with TBI. After injury, inflammation occurs as a necessary response, serving to remove or reduce challenges to the organism and subsequently restore homeostasis to promote organism survival. In an attempt to re-establish homeostasis, the inflammatory response clears foreign invaders and injured cells, enhances healing and promotes tissue repair [6,9–14]. If sustained, the inflammatory response can also become excessive creating an environment that promotes further cell death [5,15–16]. Despite the significant burden in costs and life losses associated with infection after TBI, only few studies have investigated the risk factors for development of infections in patients. In the present paper, we conducted our study to identify certain biomarkers for inflammatory reaction after TBI. The aim of the present study was to discover the differentially expressed cerebrospinal fluid (CSF) proteins and identify which can improve the clinical outcomes in TBI patients.

## Materials and methods

### TBI patients and clinical samples

One hundred and forty-five TBI patients were enrolled prospectively in this Beijing Tiantan Hospital. Medical records were retrospectively examined to detect patients who were admitted with TBI. All 145 patients acceded to cranial computerized tomography (CT) scan or brain magnetic resonance imaging (MRI) examination to clarify the diagnosis. Side of injury was evaluated based on cranial CT scan or brain MRI examination.

CSF and blood samples were obtained by trained study personnel. Those samples were taken within 24 h after the onset of TBI. Sera were isolated from peripheral blood samples, drawn from each subject before and after the expedition. Blood samples were centrifuged at 3000 rpm for 15 min. Serum samples were collected to test leukocytes. An enzyme-linked immunosorbent kit (human IL-I ELISA kit; Abcam Co.) was used for the detection of CSF interleukin-1 (IL-1).

For each patient, 1:1 sex and age-matched healthy people from the medical center at our hospital were assigned as the control group. The present study was approved by and conducted in accordance with the ethical standards of the Institutional Review Board (IRB) of the Beijing Tiantan Hospital of Capital Medical University. Informed consent was obtained by the legal authorized representative prior to study procedures.

### Protein preparation

CSF samples were milled to powder in mortar with liquid nitrogen [17–19]. Subsequently, 150 mg of powder from each sample was mixed with 1 ml of lysis buffer containing Tris-base pH = 8, 7 M Urea, 2 M Thiourea, 0.1% SDS, 2 mM EDTA, Protease inhibitor cocktail, 1 mM phenyl methyl sulfonyl fluoride) in a glass homogenizer. Homogenates were incubated on the ice for 20 min and then centrifuged at 12000***g*** for 15 min at 4°C, the supernatant was transferred to a new tube. Protein concentrations were determined by Bradford assay.

### HPLC fractionation

The first-dimension separation by micro LC was performed on a RIGOL L-3000 HPLC System (RIGOL SCIENTIFIC, INC.; Allerød, Denmark) by using a Durashell RP column (5 μm, 150 Å, 250 mm × 4.6 mm i.d., Agela). RIGOL L-3000 HPLC Systems were designed in accordance with UHPLC standard, with an outstanding operating pressure limit 9000 psi, that is the highest pressure range compared with similar HPLC system. Mobile phases A (2% acetonitrile, 20 mM NH4FA, adjusted pH to 10.0 using NH_3_.H_2_O) and B (98% acetonitrile, 20 mM NH4FA, adjusted pH to 10.0 using NH_3_.H_2_O) were used to develop a gradient elution. The solvent gradient was set as follows: 5–8% B, 2 min; 8–18% B, 11 min; 18–32% B, 9 min; 32–95% B, 1 min; 95% B, 1 min; 95–5% B, 2 min. The peptides were separated at an eluent flow rate of 1 ml/min and monitored at UV 214 nm. The column oven was set at 40°C. Eluent was collected every minute and then merged to six fractions. The samples were dried under vacuum and reconstituted in 15 μl of 0.1% (v/v) FA, 5% (v/v) acetonitrile in water for subsequent analyses [20–21].

### NanoLC-MS/MS analysis

The samples were analyzed by LC-MS/MS on a Q-Exactive HF mass spectrometer (Thermo Scientific, San Jose, CA) interfaced with EASY-nLC 1000 system at the front end. Samples were loaded into a trapping column (nanoACQUITY UPLC Symmetry C18 Trap Column, 180 µm × 20 mm, Product Number: 186006527) at a flow rate of 10 µl/min and separated with a C18 column (75 μm inner diameter, 360 μm outer diameter × 150 mm, 3 μm C18). The peptides were eluted with buffer B (0.1% formic acid in acetonitrile) gradient from 5 to 22% in 90 min at a flow rate of 350 nl/min. LC-MS/MS data were acquired using data-dependent acquisition method. Full-scan MS spectra (m/z range 300–1500) were acquired with a resolution of 60000, automatic gain control (AGC) target of 1e6, and a maximum injection time of 45 ms. MS/MS scans were acquired with a resolution of 15000, AGC target of 1e5, and maximum injection time of 100 ms. The precursor ions were selected with an isolation window of 1.2 m/z and the 20 most intense peaks with charge state 2 and above were selected for fragmentation by HCD with normalized collision energy of 30% for iTRAQ-labeled peptide. Dynamic exclusion was set to 30 s to keep the repeat sequencing of peptides to minimum [17,22].

### Protein identification and quantitation

Peptides and proteins were identified and quantitated with Sequest HT search engine using Proteome Discoverer v2.1 (Thermo Scientific) software. A standardized iTRAQ® 8plex quantification workflow module within the Proteome Discoverer was slightly modified as below and utilized for the analysis. MS/MS data were searched against the mouse SwissProt database (downloaded in September 2015; number of protein entries = 16719). The search parameters include 20 ppm precursor mass tolerance, 0.6 Da fragment mass tolerance, and trypsin miscleavage setting of two. Static modification settings included carbamidomethylation (+57.021 Da) on cysteine and iTRAQ® 8plex (304.205 Da) on N-terminus and lysine, while dynamic modifications were set to include oxidation (+15.995 Da) on methionine. Peptide spectrum matches (PSMs) were verified based on q-values set to 1% false discovery rate (FDR) using the Percolator module. Reporter Ions Quantifier node was used in the processing step workflow, and the Peptide and Protein Quantifier node was used in the consensus workflow of the Proteome Discoverer v2.1 to calculate and quantitate peptides and protein abundances and ratios across samples. Each confident protein identification was supported by at least one unique peptide. We only used ratios with *P*-values ≤0.05, and only fold changes of >1.5 were considered as significant [20,23].

### Function method description

We used three databases to predict gene functions. They were respective GO (Gene Ontology, http://www.geneontology.org), KEGG (Kyoto Encyclopedia of Genes and Genomes, http://www.genome.jp/kegg/), and COG (Clusters of Orthologous Groups). GO is an international standardization of gene function classification system. It provides a set of dynamic updating controlled vocabulary to describe genes and gene products attributes in the organism. GO has three ontologies which can describe molecular function, cellular component, and biological process, respectively. KEGG is a collection of manually drawn pathway maps representing our knowledge on the molecular interaction and reaction networks. Molecules are represented as nodes and the biological relationship between two nodes is represented as an edge [23].

### Statistics

We used Sequential Windowed Acquisition of all Theoretical fragment ions (SWATH) [19–20,22,24–28] to detect differential expression proteins in CSF in TBI patients. **S**tatistical analysis was performed using SPSS 17.0 (SPSS Inc., Chicago, IL, U.S.A.). Student’s *t* test was used for comparing the means of two separate groups. ANOVA comparison tests were used when comparing the means of multiple treatment groups with the untreated group. Statistically significant differences were defined as *P*≤0.05.

## Results

**Figure 1:**
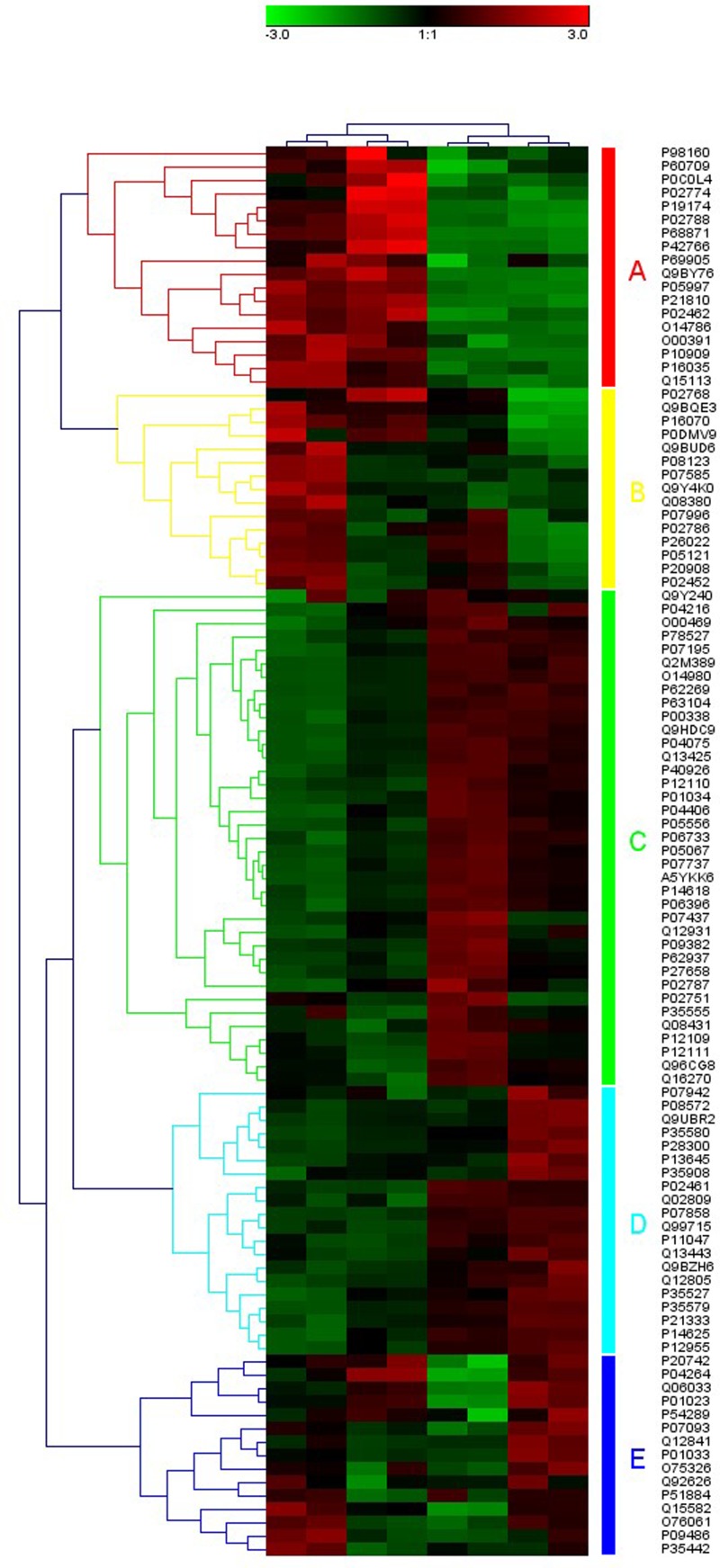
Heat maps of the 119 differentially expressed proteins clustered into groups The heat map shows the distribution of the differentially expressed proteins across all the samples, change trend, and the function of the differentially expressed proteins.

**Figure 2:**
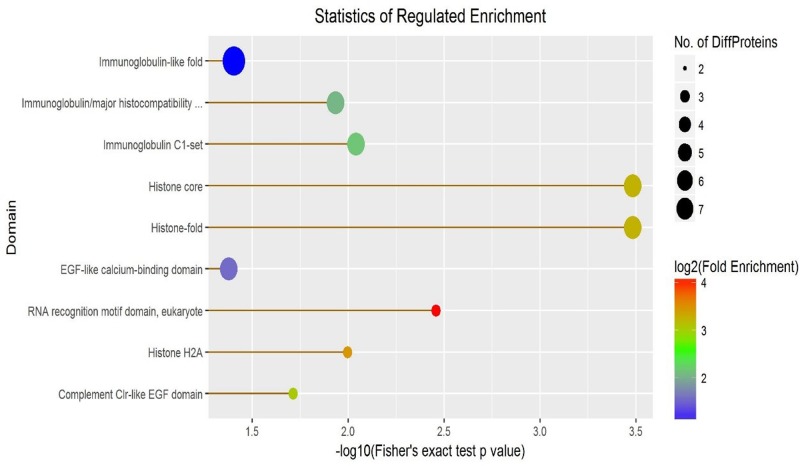
The enrichment of the differential proteins between two groups

**Figure 3:**
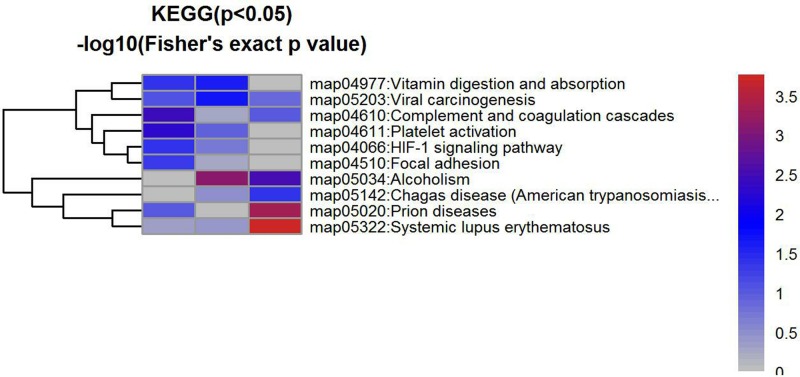
The KEGG of the differential proteins between two groups

A total of 475 proteins were identified. Of the 475 proteins, the comparison between TBI and the normal was tested. It revealed 119 proteins were changed, of which 51 proteins increased significantly and 68 proteins decreased significantly (*P*<0.05). The difference of protein abundance was more than 1.5-times and a q-value ≤0.05 indicated a significant difference between two samples. The differences were transformed by log base 2, and the expression more than 0 indicates increased expression, while expression less than 0 indicates decreased expression. The clusters of differentially expressed proteins shown in Figures 1–3.

### The overall level of peptide expression in CSF

**Figure 4:**
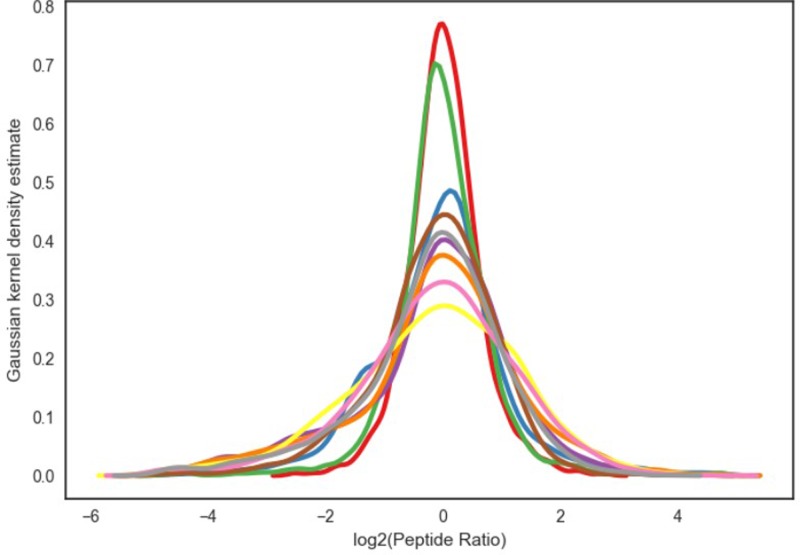
The overall level of peptide expression in CSF Multiples of the difference were transformed by log base 2. Vertical axis: the *P*-value takes the value of negative log 10.

The difference of protein abundance was more than 1.5-times and a q-value ≤0.05 indicated a significant difference between two samples. The differences were transformed by log base 2, and the expression more than 0 indicates increased expression, while expression less than 0 indicates decreased expression. The samples are of good quality and repeatability (Figure 4).

### The differential expression proteins in CSF

**Figure 5:**
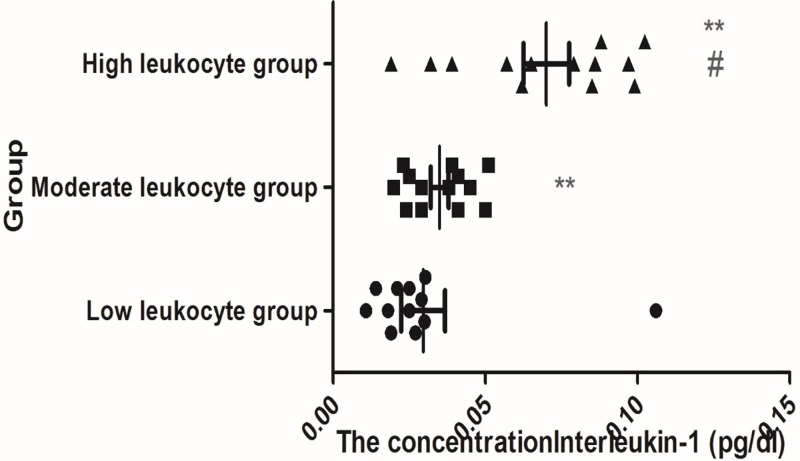
The CSF level of IL-1 in different groups ** Compared with low leukocyte group, *P*<0.001. ^#^Compared with moderate leukocyte group, *P*<0.001.

A total of 119 differentially expressed proteins were detected between samples of TBI and the normal, which were commonly expressed in all samples, indicating the differentially expressed proteins. When the patients’ Glasgow outcome score (GOS) improved, IL-1 was down-regulated, and when the patients’ GCS score deteriorated, IL-1 was up-regulated accompanied with the progression TBI (*P*<0.05). These differentially expressed proteins may be the targets of evaluating the TBI progress and prognosis which is critical to make early decision in improving the clinical outcome of TBI based on serum and CSF biomarkers, see Figure 5.

### The change in IL-1 in CSF

Compared with low leukocyte group, the level of IL-1 was increased significantly in moderate leukocyte group and high leukocyte group (*P*<0.001). Compared with moderate leukocyte group, the level of IL-1 was increased significantly in high leukocyte group (*P*<0.001), see Figure 5.

### The relationship between leukocyte and GOS

In the present study, 26 of the 29 patients with low leukocytes recovered well, 57 of the 83 patients with moderate leukocytes recovered well, and 7 of the 33 patients with high leukocytes recovered well (*X*^2^ = 29.668, *P*<0.001). Three of the 29 patients with low leukocytes were moderate disability, 7 of the 83 patients with moderate leukocyte were moderate disability, and 8 of the 33 patients with high leukocyte were moderate disability (*X*^2^ = 5.21, *P*>0.05). Zero of the 29 patients with low leukocyte were severe disability, 1 of the 83 patients with moderate leukocyte were moderate disability, and 5 of the 33 patients with high leukocyte were moderate disability (*X*^2^ = 11.361, *P*<0.001). Zero of the 29 patients with low leukocyte were vegetative state, 1 of the 83 patients with moderate leukocyte was vegetative state, and 4 of the 33 patients with high leukocyte were vegetative state (*X*^2^ = 11.389, *P*<0.001). A total of 17 of the 83 patients with moderate leukocyte died and 9 of the 33 patients with high leukocyte died (*X*^2^ = 6.221, *P*<0.001).

## Discussion

Globally, TBI is the leading cause of mortality and morbidity in children and adults under the age of 45 years [8]. It is urgent to ameliorate TBI damage and reduce the disability and case-fatality rate, TBI results from external forces, the consequence of direct impact, rapid acceleration or deceleration, a penetrating object, or blast waves from an explosion [2,8]. According to the pathology, TBI involved primary injury and secondary injury. Primary injury occurred at the initiation of the trauma, the rapid deformation of brain tissue gave rise to focal contusion, hematomas, vascular damage, and axonal injury, evolved membranolytic, cellular content efflux, hemodynamics disturbance, and neuronal necrosis. Secondary injury came after the primary injury caused a complex series of cellular and biochemical processes [8], including the blood–brain barrier (BBB) disruption, excitatory amino acid (EAA) release, free-radical generation, calcium-mediated damage, gene activation, mitochondrial dysfunction, and inflammatory responses [1,29,30]. IL-1 system plays an important role in the process of inflammation in TBI patients [9–11]. Proteins IL-1 system include the secreted agonist IL-1β, and the receptor antagonist IL-1 receptor (IL-1R) antagonist (IL-1ra), both competing for binding to the IL-1R. IL-1β and IL-1ra are highly inducible under different forms of stress, such as excitatory neurotransmitter excess occurring during seizures, in infection and inflammation and during neurotrauma [9–11,31]. The literature has been confirmed that the central nervous system (CNS) injury caused by pathogenic exposure to nerve agents results in leukocyte infiltration and macrophage activation. Chemokines guide peripheral neutrophils to damaged regions of the CNS, where up-regulation of acute phase response cytokines such as IL-1, IL-6, and tumor necrosis factor-α (TNF-α) can initiate and exacerbate a proinflammatory cascade [32,33]. IL-1 increases expression of proinflammatory mediators in microglia, suggesting IL-1 controls cytokine signaling in the CNS after injury. The literature reports that inhibiting IL-1 signaling in the IL-1R1 KO, regeneration is also being inhibited, whereas the IL-1 signaling that occurs in the IL-1Ra KO is allowing regeneration to occur over time [9]. This study showed that the absence of IL-1 signaling did not prevent this brain damage over time [9]. IL-1 is a key proinflammatory cytokine that is primarily produced in the periphery by immune cells but can also be synthesized by glia and neurons within the brain. IL-1 signaling is mediated by the binding of IL-1 a or IL-1b to the IL type one receptor (IL-1R1). IL-1 signaling can be blocked by IL-1ra which binds to the IL-1R and blocks signal transduction [9]. IL-1 is a versatile cytokine, which has unique physiological roles, including cytokine secretion in autoimmune diseases, vascular permeability, and induction of fever in sepsis [9,32,34]. Previous studies have revealed that increased IL-1 secretion enhances the expression of genes that encode proteins involved in metastasis [i.e. matrix metalloproteinases (MMPs)], and secretion of growth and angiogenic factors, including vascular endothelial growth factor (VEGF), IL-8, IL-6, TNF-α, and transforming growth factor-β [9,32–33,35].

The differentially expressed proteins in CSF include immune globulin, complement, alexin, histone, and epidermal growth factor (EGF). Those factors are the key factors of inflammatory reaction and useful for the recovery of the TBI patients. Such differential expressed proteins in CSF suggest that inflammatory marks may be the novel therapeutic targets for TBI treatment. Nonetheless, additional studies with a bigger study population should be performed to further corroborate these findings [36].

In the present study, we used SWATH [23] to detect differential expression CSF proteins in TBI patients. A total of 475 proteins were identified. Of the 475 proteins, the comparison between TBI and the normal, it revealed 119 proteins were changed, of which 51 proteins increased significantly and 68 proteins decreased significantly (*P*<0.05). The difference of protein abundance was more than 1.5-times and a q-value ≤0.05 indicated a significant difference between two samples. The differences were transformed by log base 2, and the expression more than 0 indicates increased expression, while expression less than 0 indicates decreased expression. The clusters of differentially expressed proteins shown in Figure 1, it revealed the distribution of differentially expressed proteins, the trend changes, and the putative function of the differentially expressed proteins in all samples, which may be the target genes or proteins in which we are interested (Figures 1–5). In our study, there are some major findings. (i) The leukocytes in blood samples are significantly higher; (ii) the IL-1 in CSF is significantly higher in TBI patients than in healthy; (iii) the high of IL-1 in CSF and the worse of the patient’s prognosis. Interestingly, it is well established that increased IL-1 resulting from TBI exerts a profound effect on synaptic plasticity [37]. Increasing evidence supports the complex role of microglia in response to brain injury and neurodegenerative processes [38], and have been observed to be a major source of IL-1 in the CNS following TBI [39]. Furthermore, Coughlin et al. [40] employ a radioligand to detect and map the degree of neuroinflammation associated with TBI. Caplan et al. [41] showed that the inflammatory response can also become excessive creating an environment that promotes further cell death in severe TBI. It would be feasible to utilize this radioligand in a subset of severe TBI patients and to determine whether those signal up-regulations are related to later onset of neuropsychiatric symptoms. Further study is also needed to confirm these findings and to determine whether neuroinflammation signal (such as IL-1) are related to later onset of synaptic plasticity and functional outcome in TBI.

The current study has several limitations. First, there is a relatively small sample size (*n*=154), which may cause potential bias in statistical analysis or casual statistical finding. Second, large-scale prospective studies are warranted to evaluate the prognostic contribution of leukocytes in blood samples and the level of IL-1 in CSF on clinical outcomes. Third, levels of IL-1 only tested in CSF. The blood levels of IL-1 were not obtained. Sarrafzadeh et al. [42] reported that cerebral, but not plasma IL-6, levels were predictive of the development of delayed ischemic deficits in symptomatic patients. Thus, CSF was the best sampling medium for future studies. Last, causal relationship could not be proved due to the observational study design [43]. The role of IL-1 in both the initiation and exacerbation of TBI-associated neurological deficits still need for further studies and verification of clinical trials.

## Conclusion

In summary, the differentially expressed proteins in CSF may be the novel therapeutic targets for TBI treatment. The leukocyte in blood samples and the IL-1 in CSF may be two important indicators for predicting the prognosis of TBI patients. The significance of the indicators lay in evaluating severity, monitoring progression, estimating prognosis, and guiding therapy effectively. The combined use of differentially expressed proteins and effective indicators might help in early decision-making regarding the aggressiveness of medication, potential new interventions, discharge planning, and rehabilitation.

## Abbreviations

AGC: automatic gain control
CNS: central nervous system
CSF: cerebrospinal fluid
CT: computerized tomography
FA: formic acid
GCS: Glasgow Coma Scale
GO: Gene Ontology
GOS: Glasgow outcome score
HCD: higher-energy collisional dissociation
IL-1: interleukin-1
IL-1ra: IL-1 receptor antagonist
IL-1R: IL-1 receptor
KO: knock-out
KEGG: Kyoto Encyclopedia of Genes and Genomes
MRI: magnetic resonance imaging
SWATH: Sequential Windowed Acquisition of all Theoretical fragment ion
TBI: traumatic brain injury
